# Comparing Prevalence of Sarcopenia Using Twelve Sarcopenia Definitions in a Large Multinational European Population of Community-Dwelling Older Adults

**DOI:** 10.1007/s12603-023-1888-y

**Published:** 2023-02-15

**Authors:** Anna K. Stuck, L.-T. Tsai, G. Freystaetter, B. Vellas, J.A. Kanis, R. Rizzoli, R.W. Kressig, G. Armbrecht, J.A.P. Da Silva, B. Dawson-Hughes, A. Egli, H.A. Bischoff-Ferrari

**Affiliations:** 1 Centre on Aging and Mobility, University of Zurich and City Hospital Zurich, c/o Stadtspital Waid, Tièchestrasse 99, 8037, Zürich, Switzerland; 2 Department of Aging Medicine and Aging Research, University Hospital Zurich and University of Zurich, Zurich, Switzerland; 3 Gérontopôle de Toulouse, Institut du Vieillissement, Center Hospitalo-Universitaire de Toulouse, Toulouse, France; 4 UMR INSERM 1027, University of Toulouse III, Toulouse, France; 5 Mary McKillop Institute for Health Research, Australian Catholic University, Melbourne, Australia and Centre for Metabolic Bone Diseases, University of Sheffield Medical School, Sheffield, UK; 6 Service of Bone Diseases, Geneva University Hospitals and Faculty of Medicine, Geneva, Switzerland; 7 University Department of Geriatric Medicine Felix Platter, University of Basel, Basel, Switzerland; 8 Klinik für Radiologie, Charité-Universitätsmedizin Berlin, Freie Universität Berlin and Humboldt-Universität zu Berlin, Berlin, Germany; 9 Centro Hospitalar e Universitário de Coimbra, Coimbra Institute for Clinical and Biomedical Research (iCBR), Faculty of Medicine, University of Coimbra, Coimbra, Portugal; 10 Jean Mayer USDA Human Nutrition Research Center on Aging, Tufts University, Boston, MA, USA; 11 City Hospital Waid and Triemli, University Clinic for Aging Medicine, Zurich, Switzerland

**Keywords:** Sarcopenia, aged, muscle health, prevalence study, hand strength, geriatric assessment, EWGSOP, SDOC

## Abstract

**Objectives:**

Multinational prevalence data on sarcopenia among generally healthy older adults is limited. The aim of the study was to assess prevalence of sarcopenia in the DO-HEALTH European trial based on twelve current sarcopenia definitions.

**Setting and Participants:**

This is an analysis of the DO-HEALTH study including 1495 of 2157 community-dwelling participants age 70+ years from Germany, France, Portugal, and Switzerland with complete measurements of the sarcopenia toolbox including muscle mass by DXA, grip strength, and gait speed.

**Measurements:**

The twelve sarcopenia definitions applied were Asian Working Group on Sarcopenia (AWGS1), AWGS2, Baumgartner, Delmonico, European Working Group on Sarcopenia in Older People (EWGSOP1), EWGSOP2, EWGSOP2-lower extremities, Foundation for the National Institutes of Health (FNIH1), FNIH2, International Working Group on Sarcopenia in Older People (IWGS), Morley, and Sarcopenia Definitions and Outcomes Consortium (SDOC).

**Results:**

Mean age was 74.9 years (SD 4.4); 63.3% were women. Sarcopenia prevalence ranged between 0.7% using the EWGSOP2 or AWGS2 definition, up to 16.8% using the Delmonico definition. Overall, most sarcopenia definitions, including Delmonico (16.8%), Baumgartner (12.8%), FNIH1(10.5%), IWGS (3.6%), EWGSOP1 (3.4%), SDOC (2.0%), Morley (1.3%), and AWGS1 (1.1%) tended to be higher than the prevalence based on EWGSOP2 (0.7%). In contrast, the definitions AWGS2 (0.7%), EWGSOP2-LE (1.1%), FNIH2 (1.0%) — all based on muscle mass and muscle strength — showed similar lower prevalence as EWGSOP2 (0.7%). Moreover, most sarcopenia definitions did not overlap on identifying sarcopenia on an individual participant-level.

**Conclusion:**

In this multinational European trial of community-dwelling older adults we found major discordances of sarcopenia prevalence both on a population- and on a participant- level between various sarcopenia definitions. Our findings suggest that the concept of sarcopenia may need to be rethought to reliably and validly identify people with impaired muscle health.

## Introduction

**S**arcopenia is a common disease in older adults and is associated with several adverse health outcomes including falls and fractures (1, 2). Therefore, a proper diagnosis of sarcopenia is key to implement targeted measures to eventually prevent these adverse outcomes. While sarcopenia is considered a diagnosis by the International Classification of Diseases (ICD) (3), there is still an ongoing debate on the operational definition of sarcopenia (4).

Currently, the usual clinical practice in a European setting is to apply the sarcopenia consensus definition by The European Working Group on Sarcopenia in Older People that were published in 2019 (EWGSOP2) (5). The EWGSOP2 defines sarcopenia based on both low muscle strength and low muscle mass. However, there is a controversy whether these criteria are the most feasible, reproducible, and valid to define sarcopenia. Recently, the Sarcopenia Definition and Outcome Consortium (SDOC) (6) recommended that sarcopenia be defined based on low physical performance rather than low muscle mass, in addition to low muscle strength. Moreover, there are other internationally acknowledged consensus definitions of sarcopenia that include the same sarcopenia components as EWGSOP2 (low muscle strength and low muscle mass) but apply other cut-points for these measures (7, 8, 9).

A recent meta-analysis suggests that prevalence rates differ between various sarcopenia definitions reporting a range from 5% based on the EWGSOP1 to 17% based on the definition by the International Working Group on Sarcopenia (IWGS) (4). However, this study did not report results of original studies that assessed sarcopenia prevalence based on the most recent sarcopenia definition by the SDOC. To the best of our knowledge, data among community-dwelling older participants on a multinational level comparing sarcopenia prevalence based on most current sarcopenia definitions such as EWGSOP2 and SDOC and other sarcopenia definitions are lacking. The DO-HEALTH trial provides a systematic assessment of muscle health (sarcopenia tool box) among older adults from four European countries enabling us to fill this knowledge gap.

Therefore, the aim of the study was to assess and compare prevalence of sarcopenia based on twelve internationally acknowledged consensus definitions of sarcopenia among generally healthy community-dwelling adults enrolled in the DO-HEALTH trial.

## Methods

This is an analysis of the DO-HEALTH clinical trial defined as an exploratory analysis in the study protocol (10). DO-HEALTH is a multi-centre, double-blind, randomized controlled clinical trial designed to support healthy aging in European older adults (10). The trial examined the individual and combined effects of Omega-3, Vitamin-D and Simple Home Exercise over 3 years of follow-up and is described in detail by Bischoff-Ferrari et al (10). Briefly, a total of 2157 community-dwelling men and women age 70 years and older were recruited from seven centres in five European countries, specifically Zurich, Basel, Geneva, Berlin, Innsbruck, Toulouse and Coimbra. For this study, we included data of all participants who had a valid DXA measurement at baseline for evaluation of muscle mass (n=1495).

### Consensus definitions of sarcopenia

Sarcopenia was defined using twelve definitions of sarcopenia for evaluation of sarcopenia prevalence in accordance to a recent scoping review (11) and a recent meta-analysis investigating sarcopenia definitions (25). Consequently, the following consensus definitions of sarcopenia were assessed (list in alphabetic order):

Asian Working Group on Sarcopenia 1 (AWGS1), 2010; Asian Working Group on Sarcopenia 2 (AWGS2), 2019; Baumgartner; Delmonico; European Working Group on Sarcopenia 1 (EWGSOP1), 2010; European Working Group on Sarcopenia using muscle strength of the lower extremities (EWGSOP2-LE), 2019; European Working Group on Sarcopenia in Older People 2 (EWGSOP2), 2019; Foundation for the National Institutes of Health Biomakers Consortium Sarcopenia project (FNIH1), 2014; Foundation for the National Institutes of Health Biomakers Consortium Sarcopenia project (FNIH2), 2014; International Working Group on Sarcopenia (IWGS); Morley; Sarcopenia Definitions and Outcomes Consortium (SDOC), 2020.

**Figure 1:**
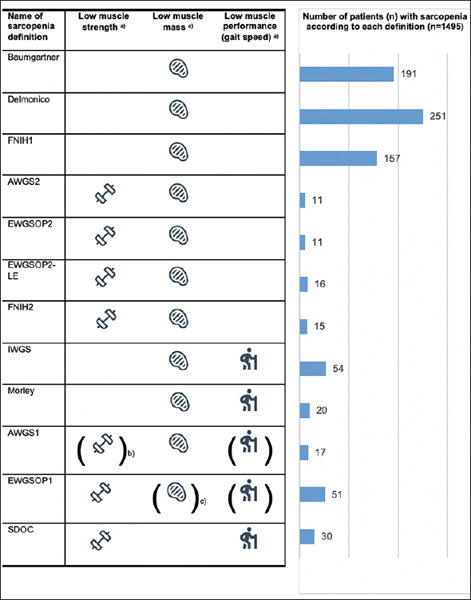
Criteria for sarcopenia definitions and number of participants with sarcopenia in the DO-HEALTH at baseline (n=1495) Abbreviations. EWGSOP, European Working Group on Sarcopenia in Older People; SDOC, Sarcopenia Definitions and Outcomes Consortium, FNIH, Foundation for the National Institutes of Health Biomarkers Consortium Sarcopenia project; IWGS, International Working Group on Sarcopenia Definition; AWGS, Asian Working Group on Sarcopenia; ASM, appendicular skeletal muscle mass, ALM, appendicular lean mass; BMI, body mass index; SPPB, short physical performance battery. a) For better readability specific cut-off values and their corresponding units for sarcopenia definitions are not displayed. b) Low muscle strength and/or low muscle performance. c) Low muscle mass and/or low muscle performance. Muscle strength of the lower extremities was assessed by the repeated chair stands test. Participants were instructed to perform 5 repeats of the sit-to stand chair test using manual timing.

Sarcopenia components of all consensus definitions and their corresponding cut-offs are summarized in Figure 1.

### Assessment of sarcopenia

Components of sarcopenia (low muscle strength, low muscle mass, low physical performance) were measured at baseline (10).

Muscle strength of the upper extremities was measured using the grip strength test. Participants were asked to firmly squeeze a large-sized balloon of the Martin vigorimeter. The maximum of three consecutive measurement trials at the dominant hand was used for analysis. Conversion of grip strength in kPA based on the Martin vigorimeter into kilograms was performed in accordance to Neumann et al. (12) by applying the conversion factor of 0.61.

Total appendicular skeletal muscle mass (expressed as ASMMI) was measured in four study sites France, Germany, Portugal, and Switzerland (Zurich, Lunar iDXA, GE-Healthcare) using DXA measurement. All DXA evaluations were performed centrally at the DXA QA and Central Reading Center Berlin to increase comparability between study sites.

Gait speed was measured as part of the short physical performance battery on a 4-meter walk test including distance for acceleration and deceleration using manual timing (stopwatch). Participants were asked to perform the walking test at their usual gait speed and were allowed to use their usual walking aid. The higher gait speed of two trials was used for analysis.

Healthy aging was assessed based on the Nurses' Health Study definition meeting all four criteria: (1) no major chronic disease, (2) no disabilities, (3) no cognitive impairment (Montreal Cognitive Assessment MoCA≥25 points), (4) no mental health limitation (5-item Geriatric depression scale <2, and no diagnosis of depression). Participants who did not fulfill these criteria were classified as non-healthy agers.

### Statistical analysis

Clinical characteristics of the study population are described overall, and by sex. Normally distributed continuous variables are presented as mean and standard deviation (SD) and non-normally distributed variables as median and interquartile range (IQR). Categorical variables are presented in frequencies and percentages. Overall prevalence is calculated based on the 12 sarcopenia definitions. Moreover, prevalence of sarcopenia was calculated for predefined subgroups: Sex (female vs. male) (13); age (70–74 years vs. 75+ years); country (14) (Switzerland, Germany, France, Portugal); BMI (<25 or ≥25 kg/m^2^) (15). Based on a prior study showing a link between healthy aging and physical function (16) we further analyzed the subgroup of healthy vs. non-healthy agers as defined in the Methods section. Analyses were performed using SAS version 9.4 (SAS Institute, Cary, NC) with a fixed 5% significance level.

## Results

Of 1495 participants, mean age was 74.9 (4.4) years and 63.3% were women. Clinical characteristics of participants are displayed in Table 1. Overall, 551 participants (36.8%) were living in Switzerland, 347 (23.2%) in Germany, 300 (20.1%) in Portugal, and 297 (19.9%) in France. Overall, 45 participants (3%) had low grip strength, and 155 (10.4%) low muscle mass according to the cut-off definitions by EWGSOP2. 164 (11.0%) had low gait speed (<0.8m/sec) based on the criteria by SDOC and EWGSOP1. Over one third of participants (n=529, 35.4%) was classified as non-healthy agers.

**Table 1:** Clinical characteristics of the participants (n=1495)

	**Overall**	**Women**	**Men**
	**n=1495**	**n= 946**	**n=549**
Age, years, mean (sd)	74.9 (4.4)	74.8 (4.4)	75.2 (4.4)
BMI, kg/m^2^, mean (sd)	26.6 (4.3)	26.5 (4.7)	26.8 (3.5)
Women, n (%)	946 (63.3)		
Country, n (%)			
-France	297 (19.9)	179 (18.9)	118(21.5)
-Portugal	300 (20.1)	192 (20.3)	108 (19.7)
-Germany	347 (23.2)	245 (25.9)	102 (18.6)
-Switzerland	551 (36.8)	330 (34.9)	221 (40.3)
Living alone, n (%)	614(41.1)	505 (53.4)	109 (19.8)
Education, years, mean (sd)	12.4 (4.5)^a^	11.8 (4.3)^b^	13.5 (4.6)
Comorbidity scoree), median (IQR)	3 (1; 5)^a^	3(1; 6)	2 (0; 4)^d^
Number of medications, mean (sd)	3.4 (2.9)	3.5 (3.0)	3.3 (2.7)
SPPB score, median (IQR)	11 (10; 12)^a^	11 (10; 12)^f^	11 (10; 12)^s^
Prior fall, n (%)	604 (40.4)	426 (45)	178 (32.4)
Grip strength, kPa, mean (sd)	58.6 (17.9)^a^	49.8(11.6)	73.8 (16.8)^i^
Low grip strength, n (%)^b^	45 (3.0)^a^	24 (2.5)	21 (3.8)^p^
Appendicular skeletal muscle mass, kg/m^2^, mean (sd)	7.15(1.1)	6.6 (0.9)	8 (0.9)
Low muscle mass, n (%)^c^	155 (10.4)	83 (8.8)	72(13.1)
Gait speed, *ml* sec, mean (sd)	1.15(0.3)^l^	1.13 (0.2)^m^	1.2 (0.2)^n^
Low physical performance, n (%)^d^	164(11.0)^a^	128 (13.5)^r^	36 (6.6)^s^

Abbreviations: SD (standard deviation), BMI (Body Mass Index), SPPB (Short physical performance battery); IQR (interquartile range); EWGSOP2, European Working Group on Sarcopenia in Older People 2; EWGSOP1, European Working Group on Sarcopenia in Older People 1; SDOC, Sarcopenia Definitions and Outcomes Consoritum; a) n=1493 (n=2 missing): b. Low grip strength defined according to EWGSOP2 as grip strength <16kg for women, and <27kg for men; c. Low muscle mass defined according to EWGSOP2 as muscle mass <5.5 kg/m^2^ for women, and <7.0kg/m^2^ for men; d. Low physical performance defined according to SDOC and EWGSOP1 as gait speed <0.8m/sec; e. Comorbidity score by self-administered questionnaire

Prevalence of sarcopenia ranged between 0.7% (n=11) based on both EWGSOP2 and AWGS2 definitions, and 16.8% (n=251) based on definition by Baumgartner (Figure 1). Thereby, the prevalence based on sarcopenia definitions by AWGS1, Baumgartner, Delmonico, EWGSOP1, FNIH1, IWGS, Morley and SDOC tended to be higher than the prevalence based on EWGSOP2. In contrast, prevalence of sarcopenia based on the definitions by AWGS2, EWGSOP2-LE, and FNIH2 tended not to differ from that assessed by EWGSOP2.

Figure Figure 2 Panel A, Figure 2 Panel B, Figure 2 Panel C, Figure 2 Panel D, Figure 2 Panel E depicts the level of agreement on a participant level between the three most recent sarcopenia definitions (EWGSOP2, AWGS2, SDOC) by Venn plot. Among participants categorized as sarcopenic either by EWGSOP2 or SDOC definition (n=39), agreement of sarcopenia definitions was observed in only two participants. The EWGSOP2 and AWGS2 definitions identified precisely the same individuals (n=11) as being sarcopenic. Venn plot diagrams comparing agreement between other combinations of sarcopenia definitions are shown in Figure 2 Panels B-E.

**Figure 2 Panel A:**
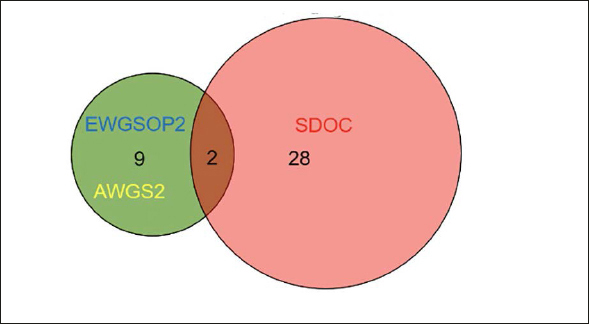
Venn plot comparing the three most recent sarcopenia definitions (EWGSOP2, AWGS2, SDOC) for sarcopenia diagnosis displaying absolute numbers of patients (n)

**Figure 2 Panel B:**
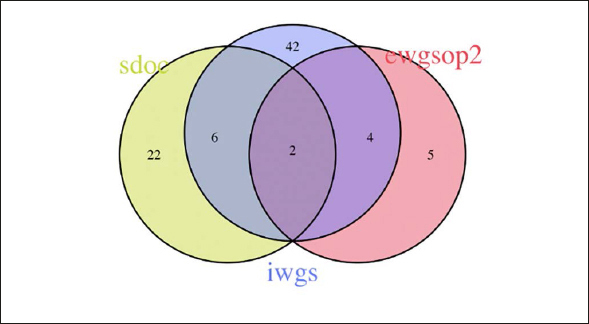
Venn plot comparing different criteria combinations using two criteria for sarcopenia diagnosis (SDOC=low grip and low gait; IWGS= low mass and low gait; EWGSOP2=low mass and low grip) displaying aboluate numbers of patients (n)

**Figure 2 Panel C:**
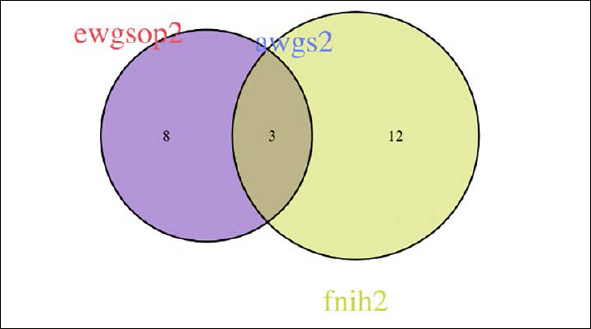
Venn plot comparing different sarcopenia definitions all based on low grip strength and low muscle mass (EWGSOP2, AWGS2, FNIH2) for sarcopenia diagnosis displaying absolute numbers of patients (n)

**Figure 2 Panel D:**
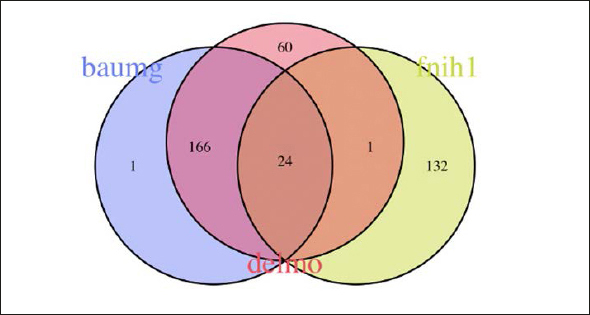
Venn plot comparing different sarcopenia definition all based on low muscle mass only (Baumgartner, Delmonico, FNIH1) for sarcopenia diagnosis displaying absolute numbers of patients (n)

**Figure 2 Panel E:**
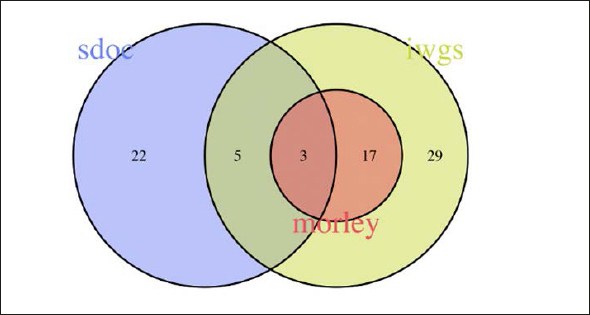
Venn plot comparing different sarcopenia definitions all based on low gait speed and either low grip strength (SDOC) or low muscle mass (Morley, IWGS) for sarcopenia diagnosis displaying absolute numbers of patients (n)

Tables Table 2A, Table 2C summarizes the results of subgroup analyses. Similar to results in the total study population, four out of the eleven definitions (AWGS1, EWGSOP2-LE, FNIH2, and Morley) showed higher agreement with EWGSOP2 and AWGS2 in terms of identifying individuals with sarcopenia. Notably, sarcopenia prevalence between EWGSOP2 and SDOC significantly differed among women (0.4% vs. 2.0%), but not in men (1.3% vs. 2.0%). Similarly, sarcopenia prevalence was higher using SDOC compared to EWGSOP2 among participants aged 75 years and older (1.4% s. 3.9%), but not in younger participants (0.2% vs. 0.6%). The same pattern was observed in obese (0.1% EWGSOP2 vs, 2.3% SDOC), versus non-obese participants (1.8% EWGSOP2 vs. 1.4% SDOC). Table 2B displays the difference between sarcopenia definition in each of the four countries. Finally, in the subgroup of non-healthy agers, sarcopenia prevalence was higher using SDOC compared to EWGSOP2 (2.8% vs. 1.1%) vs. but did not differ in the subgroup of healthy agers (0.2% vs. 0.2%) (Table 2C).

**Table 2A:** Prevalence of sarcopenia based on 12 sarcopenia definitions by sex, age, and BMI^a)^ (n=1495)

	**By**	**sex**	**By age**		**By BMI**	
	**Men**	**Women**	**70–74 years**	**≥75 years**	**<25kg/m**^**2**^	**≥25kg/m**^**2**^
	**(n=549)**	**(n=946)**	**(n=850)**	**(n=645)**	**(n=554)**	**(n=941)**
	**n (%)**	**n (%)**	**n (%)**	**n (%)**	**n (%)**	**n (%)**
Baumgartner	118 (21.5)	73 (7.7)	105 (12.3)	86 (13.3)	161 (29.1)	30 (3.2)
Delmonico	117 (21.3)	134 (14.2)	141 (16.6)	110 (17)	217 (39.2)	34 (3.6)
FNIH1	93 (16.9)	64 (6.8)	64 (7.5)	93 (14.4)	14 (2.5)	143 (15.2)
AWGS2	7 (1.3)^b^	4 (0.4)	2 (0.2)	9 (1.4)^h^	10 (1.8)^n^	1 (0.1)?
EWGSOP2	7 (1.3)^b^	4 (0.4)	2 (0.2)	9 (1.4)^h^	10 (1.8)^n^	1 (0.1)o
EWGSOP-LE	7 (1.3)^c^	9 (1)^d^	7 (0.8)^i^	9 (1.4)^j^	13 (2.4)^p^	3 (0.3)^q^
FNIH2	9 (1.6)^b^	6 (0.6)	2 (0.2)	13 (2)^h^	3 (0.5)^n^	12 (1.3)^o^
IWGS	32 (5.8)^e^	22 (2.3)^f^	15 (1.8)^k^	39 (6.1)^l^	41 (7.4)	13 (1.4)^r^
Morley	14 (2.5)^e^	6 (0.6)^f^	3 (0.3)^k^	17 (2.6)^l^	17 (3.1)	3 (0.3)^r^
AWGS1	10 (1.8)	7 (0.7)	2 (0.2)	15 (2.3)	14 (2.5)	3 (0.3)
EWGSOP1	16 (2.9)^b^	35 (3.7)	13 (1.5)	38 (5.9)^h^	28 (5.1)^n^	23 (2.4)^o^
SDOC	11 (2.0)^g^	19 2.0)^f^	5 (0.6)^k^	25 3.9)^m^	8 (1.4)^n^	22 (2.3)^s^

a. Subgroups as defined in Methods section b. N=547; c. N=541; d. N=936; e. N=548; f. N=945; g. N=546; h. N=643; i. N=846; j. N=631, k. N=849; l. N=644; m. N=642; n. N=553; o. N=940; p. N=550; q. N=927; r. N=939; s. N=938

**Table 2C:** Prevalence of sarcopenia based on twelve sarcopenia definitions by the subgroup of healthy agers ^a)^ (n=1495)

**Name of definition**	**Healthy agers (n=936) n (%)**	**Non-healthy agers (n=529) n (%)**
Baumgartner	78 (14.7)	106 (11.3)
Delmonico	103 (19.5)	140 (15.0)
FNIH1	21 (3.4)	132 (14.1)
AWGS2	1 (0.2)	10 (1.1)^b^
EWGSOP2	1 (0.2)	10 (1.1)^b^
EWGSOP2-LE	5 (0.9)^c^	9 (1)^d^
FNIH2	0 (0)	15 (1.6)^b^
IWGS	13 (2.5)^c^	37 (4.0)^b^
Morley	5 (1.0)^c^	11 (1.2)^b^
AWGS1	2 (0.4)	13 (1.4)
EWGSOP1	7 (1.3)	39 (4.2)^b^
SDOC	1 (0.2)^c^	26 (2.8)^e^

Abbreviations: EWGSOP2-LE, European Working Group on Sarcopenia in Older People based on muscle strength of lower extremities; SDOC, Sarcopenia Definitions and Outcomes Consortium, FNIH, Foundation for the National Institutes of Health Biomakers Consortium Sarcopenia project; IWGS, International Working Group on Sarcopenia Definition; Ref, Referent; a. Healthy vs. non-health agers defined in Methods section; b) N=935; c) n=528; d) n=921; e) n=934

**Table 2B:** Prevalence of sarcopenia based on twelve sarcopenia definitions by country ^a)^ (n=1495)

**Name of Definition**	**France (n=297) n (%)**	**Germany (n=347) n (%)**	**Portugal (n=300) n (%)**	**Switzerland (n=551) n (%)**
Baumgartner	54 (18.2)	47 (13.5)	19 (6.3)	71 (12.9)
Delmonico	70 (23.6)	63 (18.2)	20 (6.7)	98 (17.8)
FNIH1	35 (11.8)	13 (3.7)	75 (25)	34 (6.2)
AWGS2	1 (0.3)	4 (1.1)	1 (0.3)	5 (0.9)^b^
EWGSOP2	1 (0.3)	4 (1.1)	1 (0.3)	5 (0.9)^b^
EWGSOP2-LE	9 (3.1)^c^	1 (0.3)	5 (1.7)^d^	1 (0.2)^b^
FNIH2	1 (0.3)	1 (0.3)	12 (4)	1 (0.2)^b^
IWGS	16 (5.4)^e^	3 (0.9)	7 (2.3)	28 (5.1)
Morley	8 (2.7)^e^	1 (0.3)	2 (0.7)	9 (1.6)
AWGS1	2 (0.7)	4 (1.1)	2 (0.7)	9 (1.6)
EWGSOP1	11 (3.7)	7 (2.0)	19 (6.3)	14 (2.5)^b^
SDOC	4 (1.4)^e^	0 (0)	17 (5.7)	9 (1.6)^b^

a. Subgroups as defined in Methods section; b. N=549; c. N=289; d. N=292; e. N=295

## Discussion

Our study describes and compares prevalence of sarcopenia between EWGSOP2 and various sarcopenia definitions among community-dwelling older participants from four European countries (France, Germany, Portugal and Switzerland). We found that the sarcopenia prevalence based on EWGSOP2 tended to be lower than prevalence by most sarcopenia definitions such as SDOC with overlap only in a minority of participants.

Overall, there is a considerable number of previous studies showing heterogeneity of sarcopenia prevalence applying different sarcopenia definitions (17, 18). However, in the last three years new sarcopenia definitions were elaborated and data on comparison of sarcopenia prevalence based on the most recent sarcopenia definitions (e.g. SDOC, EWGSOP2) and on an individual participant-level are scarce. Therefore, to the best of our knowledge, the results of the present study fill these gaps of knowledge.

Generally, we found in this sample of older, generally healthy community-dwelling participants, a low prevalence of sarcopenia (0.9%), which is similar to results from a population-based cohort of the Canadian Longitudinal Study on Aging (0.2%) (19). However, prevalence rates of prior studies vary substantially (20). For example, Sousa Santos et al. (21) described a sarcopenia prevalence of 4.4% based on EWGSOP2 criteria among older adults from Portugal. Similarly, Chew et al. (22) described a prevalence of sarcopenia (4%) using EWGSOP2 criteria among older adults from Korea. In contrast, Jyvakorpi et al. (23) reported a prevalence of 20.8% among old men living in Finland.

We further found that certain definitions (such as SDOC) tended to have a higher prevalence than EWGSOP2 and AWGS2 both based on muscle mass and grip strength. Moreover, we found that on a participant-level, agreement between these definitions was low. While studies almost consistently reported, that applying EWGSOP2 compared to EWGSOP1 results in a substantial mismatch of case-finding (24), studies comparing prevalence of sarcopenia on an individual patient-level based on SDOC vs. EWGSOP2 are scarce. Harvey et al. (25) described a prevalence of 2.9% based on EWGSOP2 compared to 1.0% using the SDOC definition in a cohort among men in Sweden, United States, and Hong Kong. However, this study only refers to data in male patients.

There may be several factors explaining these differences of prevalence and patient-level discordances between sarcopenia definitions. First, sarcopenia definitions themselves vary in terms of components that are included in the definition of sarcopenia. While SDOC is based on low gait speed and low grip strength, EWGSOP2 defines sarcopenia as low muscle mass and low grip strength instead. These various criteria being used for definition of sarcopenia reflect the ongoing debate what parameters of the muscle do most validly reflect the sarcopenic state. Moreover, although the SDOC and EWGSOP share the criterion of low grip strength in their definitions of sarcopenia, the cut-off for low grip strength by the EWGSOP2 compared to SDOC is lower for both women (<16 kg vs. <20kg) and men (<20 kg vs. <35.5kg), consequently resulting in lower prevalence of sarcopenia when applying the EWGSOP2 cut-off value for low grip strength definition instead of the SDOC definition. A previous study similarly demonstrated that the choice of cut-off values for grip strength has a substantial impact on the proportions identified with sarcopenia and frailty (26). Van Ancum even concluded that the lower cut-off point for grip strength by the EWGSOP2 resulted in fewer older adults being diagnosed with sarcopenia (13).

Our study has several strengths. We were able to analyse data from the largest European study investigating community-dwelling older adults. The prevalence of sarcopenia has been evaluated at baseline according twelve internationally acknowledged definitions of sarcopenia. Standardized protocols for assessment of sarcopenia components (muscle strength, muscle mass, and physical performance) were used which ensures reproducibility of measurements. Also, we were able to analyse concordance of sarcopenia definitions on an individual participant-level. Moreover, sarcopenia components were assessed using the most valid device and methodological approach: muscle strength using grip strength and repeated chair stands test, muscle mass using DXA, and physical performance using gait speed test.

There are several limitations to this study. First, DO-HEALTH is a sample of selected community-dwelling older adults, who are generally healthy. Thus, results of our study cannot be generalized to other clinical settings or populations. Based on low proportions of participants with sarcopenia, it is not appropriate to analyze and compare predictive validity of sarcopenia definitions on clinical outcomes over follow-up. Second, our study focus is on comparison of sarcopenia prevalence of various sarcopenia definitions, and cannot recommend a specific sarcopenia definition. Third, we selected commonly used and internationally acknowledged consensus definitions of sarcopenia comparing them to the current European standard of practice, the EWGSOP2 consensus definition. However, our results may not apply to other definitions of sarcopenia using other cut-off values of low muscle strength, low muscle mass, or low physical performance, respectively (26). Fourth, we put the focus on description of prevalence applying various consensus definitions, but there may be other additional factors having an impact on prevalence of sarcopenia. For example, the choice of the device to measure grip strength, the approach to measure gait speed, and the method of measuring muscle (DXA or bioelectrical impedance analysis or D3-Creatine dilution (27)) mass may play a role, as well. Finally, it was an a priori decision based on evidence to investigate prevalence in predefined subgroups of age, sex, BMI, country, and healthy aging. However, due to limited size of subgroups, findings from subgroup analyses have to be interpreted with caution.

Our study has several implications for clinical research and practice. Based on the finding that sarcopenia prevalence greatly among sarcopenia definitions, it is important, that medical doctors who are in charge of diagnosing sarcopenia recognize the fact that diagnosis of sarcopenia largely depends on the sarcopenia definition they apply for their individual participant. This aspect is all the more important, because the diagnosis or non-diagnosis of sarcopenia impacts the decision on therapeutic measure for a patient, ultimately also affecting clinical outcomes of a patient. Moreover, our findings among subgroups of participants suggest that the choice of sarcopenia definition may have a particular impact on sarcopenia diagnosis in distinct subgroup of participants. Specifically, women, aged, obese, non-healthy agers and participants from Portugal may be most prone to be affected by the choice of the sarcopenia definition. As a next step, observational studies including these vulnerable subgroups are needed to compare predictive validity of sarcopenia definitions on key clinical outcomes such as falls and fractures also including women (25). This will eventually support advances in the field to agree on most valid criteria and corresponding cut-off values to define sarcopenia.

Future efforts on advancing our understanding of current discrepancies between existing sarcopenia definitions may need to include a stronger link to pathophysiological changes reflecting micro- and macrostructural changes of muscle (28). This may be achieved by an objective measure of structural changes of the muscle by a low-cost point-of-care ultrasound (29) or a costly assessment by MRI (30). Additionally, a standardized validation effort of current sarcopenia definitions with regard to a core set of clinical outcome measures relevant to sarcopenia may help identify the most valid definition, which may differ by outcome assessed (11).

In conclusion, we found a generally low prevalence of sarcopenia in this multinational European sample of overall healthy community-dwelling participants. There was a major discordance between sarcopenia definitions identifying sarcopenia both on a population- and on an individual participant-level. Our findings suggest that the concept of sarcopenia may need to be rethought to reliably and validly identify people with impaired muscle health.
